# Hu’po Anshen Decoction Accelerated Fracture-Healing in a Rat Model of Traumatic Brain Injury Through Activation of PI3K/AKT Pathway

**DOI:** 10.3389/fphar.2022.952696

**Published:** 2022-07-18

**Authors:** Jing Shen, Yan-Ze Li, Sai Yao, Zhou-Wei Zhu, Xiang Wang, Hui-Hui Sun, Wei-Feng Ji

**Affiliations:** ^1^ Department of Orthopedics, The First Affiliated Hospital of Zhejiang Chinese Medical University, Hangzhou, China; ^2^ Department of Neurology, The First Affiliated Hospital of Zhejiang Chinese Medical University, Hangzhou, China; ^3^ Department of Orthopedics and Traumatology of Traditional Chinese Medicine, Zhejiang Chinese Medical University, Hangzhou, China; ^4^ Department of Orthopaedics, LanXi People's Hospital, Jinhua, China

**Keywords:** fracture healing, hupo anshen decoction, traumatic brain injury, UPLC/Q-TOF MS, metabolomics

## Abstract

Hu’po Anshen decoction (HPASD) is a traditional Chinese medicine formula comprising five herbal medicines for the treatment of concussion and fracture healing, but its pharmacological mechanism is still unclear. Ultra-performance liquid chromatography coupled with quadrupole time of flight mass spectrometry (UPLC/Q-TOF MS) was used to analyze the main active components of HPASD. Rats were randomly assigned to fracture group, fracture combined with traumatic brain injury (TBI) group (FBI) and FBI combined with HPASD treatment group (FBIH). Rats in the FBIH group were given oral doses of HPASD (2.4 g/kg, 4.8 g/kg and 9.6 g/kg) for 14 or 21 consecutive days. The fracture callus formation and fracture sites were determined by radiographic analysis and micron-scale computed tomography (micro-CT) analysis. Hematoxylin and eosin (H&E) staining and a three-point bending test were applied to assess histological lesions and biomechanical properties, respectively. The levels of cytokines-/protein-related to bone formation and differentiation as well as PI3K/AKT pathway-related proteins were determined by Enzyme-linked immunosorbent assay (ELISA), quantitative reverse transcription-polymerase chain reaction (qRT-PCR), or western blot assays, respectively. UPLC-Q/TOF-MS-based serum metabolomic analysis was also performed to investigate the therapeutic effects of HPASD in the treatment of FBI. UPLC/Q-TOF MS analysis showed the chemical components in HPASD, including flavonoids, amino acids, saponins, and phenylpropanoid constituents, etc. HPASD dose-dependently promoted callus formation, increased bone density, improved mechanical parameters and morphological scores, and facilitated the expressions of VEGF, PDGF, bFGF, VEGFA, CoL1A1, RUNX2, BMP2, and Aggrecan, inhibited the expression of MMP13, and activated PI3K/AKT pathway. Metabolomics analysis revealed abnormalities of malate-aspartate shuttle and glucose-alanine. HPASD accelerates fracture healing by promoting bone formation and regulating the malate-aspartate shuttle and glucose-alanine cycle, which might be associated with the activation of the PI3K/AKT pathway.

## Introduction

Fracture is a common clinical disease and the leading cause of death and disability in the world (Borgström et al., 2020). In the treatment of fracture, delayed union or even nonunion of fracture seriously affects the prognosis of fracture (Einhorn and Gerstenfeld, 2015). Clinical findings showed that compared with patients with simple fractures, patients with fractures combined with traumatic brain injury (TBI) had more callus, faster fracture healing and even heterotopic ossification (Hofman et al., 2015). However, the specific mechanism of brain injury accelerating fracture healing is still unclear. Therefore, exploring the potential mechanisms behind this phenomenon to provide new ideas for the treatment of fractures has become one of the hot spots in the research field of bone injury.

Traditional Chinese medicine (TCM) has some advantages in the treatment of TBI and fracture due to its multi-targets, low cost and fewer side effects (Mukwaya et al., 2014; Yang et al., 2016). Hu’po Anshen decoction (HPASD), a clinically used to treat concussions and promoted the healing of distal radius fractures in China, was proposed by the deceased professor Yinhua Lu (The First Affiliated Hospital of Zhejiang Chinese Medical University, Zhejiang, China). HPASD consists of five herbs: amber (Hupo), Fossilia Dentis Mastodi (Longchi), Cinnabaris (Chensha), chrysanthemum (Juhua), and mulberry leaf (Sangye), among which amber (Zhu et al., 2019), Dens Draconis (Oguri et al., 2017) and Cinnabar (Liu J. et al., 2018) have the functions of tranquilizing the mind, while Chrysanthemum (Yuan et al., 2020) and Mori folium (Joh et al., 2015) have the functions of harmonizing yin and yang. However, the pharmacological mechanism of HPASD in the treatment of TBI combined with a fracture is still unclear.

Due to the complex components and diverse structures of TCM, the analysis, identification and content determination of its active components have become difficult. Recently, ultra-performance liquid chromatography coupled with quadrupole time of flight mass spectrometry (UPLC/Q-TOF MS) technology can be used for the identification of TCM components (Sang et al., 2021), which is convenient for finding the active components of TCM and clarifying the pharmacological mechanism of TCM. In addition, UPLC/Q-TOF MS-based metabolomics can also provide new insights into the therapeutic effects of TCM (Qin et al., 2018).

**FIGURE 1:**
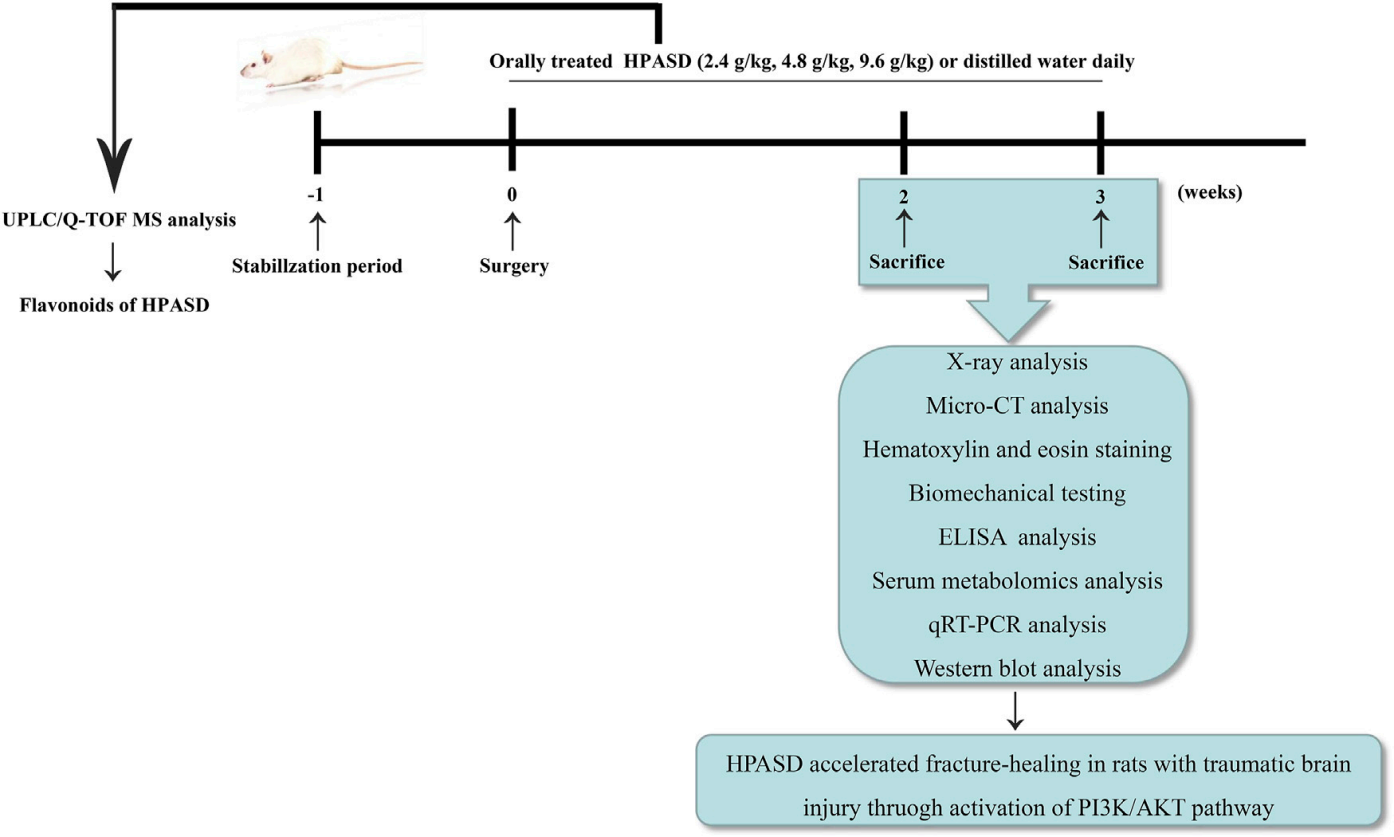
The workflow of the pharmacological study of HPASD.

In this study, the active ingredient of HPASD was firstly analyzed by UPLC/Q-TOF MS, and the pharmacological mechanism of HPASD in the treatment of TBI combined with fracture was determined through the animal model and UPLC/Q-TOF MS-based metabolomics. Schedule of experimental design to demonstrate the effect of HPASD on fracture-healing in a rat model of traumatic brain injury was shown in Figure 1.

## Materials and Methods

### Composition and Preparation of HPASD

The HPAS decoction contained 3 g amber (Hupo), 15 g of fossilia dentis mastodi (Longchi), 3 g of cinnabaris (Chensha), 10 g of chrysanthemum (Asteraceae; Chrysanthemum × morifolium (Ramat.) Hemsl), and 15 g of mulberry leaf (Moraceae; Morus alba L). All the herbs were obtained from were Binjiang out-patient department of Zhejiang Chinese Medical University (Zhejiang, China) and were fully validated by Pro. Xiao-feng Yuan (Medical Research Institute of Traditional Chinese Medicine, Zhejiang Chinese Medical University, Zhejiang, China) according to the Chinese Pharmacopoeia 2015. The 46 g medicinal herb mixture was soaked in 2 L of water for 30 min, boiled for 60 min to obtain aqueous extracts, then the extraction was filtered and concentrated to 0.48 g crude drug/mL as HPASD for the experiments and stored at -20°C before use.

### UPLC-Q/TOF-MS Analysis

0.5 ml HPASD solution and 0.5 ml of methanol were mixed and centrifuged at 14000r/min for 20 min to obtain the supernatant which was separated on the ACQUITY UPLC BEH C18 column (2.1 × 100 mm, 1.7 μm, Waters, United States) at 40°C. The mobile phase included 0.1% formic acid acetonitrile (A) and 0.1% formic acid water (B). Gradient elution with a flow rate of 0.3 ml/min was performed as follows: 0–12 min, 99%B-70%B; 12–14 min, 70%-50% B; 14–17 min, 50%-10% B; 17–19 min, 10%-1% B; 19–19.1 min, 1%-99% B; 19.1–22 min, 99%B. Q-TOF-MS analysis was carried out in positive and negative ion modes on SCIEX X-500R Quadrupole Time-of-Flight Mass Spectrometer (AB SCIEX, United States) equipped with TurboIonSpray ion source (AB SCIEX, United States). The screening of target substances without standard substances was completed by searching TCM MS/MS Library (containing more than 1,000 kinds of secondary data of Chinese medicinal compounds) configured by SCIEX OS.

### Animals and Ethics Statement

A total of 72 male Sprague Dawley rats (12 weeks, 400–420 g) were supplied by Shanghai Slake Animal Laboratory Co. Ltd. (Certificate No. SCXK (Hu) 2017–0005) and housed in an environment with room temperature maintained at 22 ± 2°C, humidity maintained at 50–60% and lighting set to 12/12 h light/dark cycle. Our research was approved by the Ethics Committee of Zhejiang Eyong pharmaceutical research and development co, ltd. (Certificate No. SYXK (Zhe) 2020–0024, Hangzhou, China).

### Establishment of Brain Injury Model

The brain injury model was established as previously described with minor modification (Chen et al., 2018). After the rats were anesthetized with pentobarbital sodium (50 mg/kg), the rats were fixed, and the hairs in the 2 cm × 2 cm area of the top of the rat’s brain were removed. Under aseptic conditions, the scalp of the top of the rat’s brain was cut, and the right parietal bone of the rat was fully exposed and the periosteum was separated. A bone window with a diameter of about 5 mm was drilled at the midline adjacent to the coronal suture without disturbing the dura. A weight weighing 20 g falls freely from a height of 30 cm along the guide rod and hits the cone to cause moderate brain injury in rats. The scalp was sutured.

### Establishment of Fracture Model

After the rats’ vital signs were stable after brain injury and anesthesia, the rats were fixed on the operating table to undergo fracture surgery as described in detail previously (Huang et al., 2015; Zhu et al., 2021). Briefly, an incision of the skin was made along the front edge of the left tibia. The anterior tibial muscle was bluntly separated. The middle part of the tibia (about 2.0 cm below the knee joint) was sawed off from the lateral side of the tibia with a pendulum saw (the saw blade thickness was 1.0 mm), and then a K-wire (1.0 mm in diameter) was used for retrograde fixation in the medullary cavity. After the operation, 40,000 units of penicillin were injected intramuscularly into the rats for three consecutive days, and the rats were placed in a cage for free food and activity.

### Groups and Drug Treatment

The rats were randomly divided into six groups, the control group, fracture-only group, fracture + TBI group, and fracture + TBI + HPASD groups. Rats in the fracture-only group received only fracture management, while rats in the fracture + TBI group received both fracture and brain injury management. Rats in the fracture + TBI + HPASD groups were intragastrically administered with a low dose of HPASD (2.4 g crude drug/kg bodyweight), a medium dose of HPASD (4.8 g crude drug/kg bodyweight, and a high dose of HPASD (9.6 g crude drug/kg bodyweight) once a day for 14 or 21 consecutive days at 1 ml per 100 g body weight after treatment for bone fractures and brain injuries, respectively. The medium dose of HPASD for fracture + TBI rats was calculated with a body surface area normalization method according to the normal clinical dose (Reagan-Shaw et al., 2008) using the following formula: Rats (g/kg) = [human dose (46 g crude drug/day)/human weight (60 kg)]×6.3. In this study, the low-dose and high-dose HPASD are used as 1/2 and 2 times medium-dose of HPASD, respectively. Rats in the control group were exposed only to the tibia but did not amputate the tibia and were not treated for brain damage. Additionally, rats in the control group, fracture-only group, and fracture + TBI group received the same volume of water daily for 14 or 21 days via oral gavage.

### Radiographic Analysis of Fracture Callus Formation

On the 14th or 21st day after administration, X-ray films of the bones of rats in each group were obtained by X-ray equipment (Philips, Germany).

### Micro-CT Analysis of the Fracture Sites

On the 14th or 21st day after administration, a micro-CT scan with 10 μM resolution (Viva CT 40, Scanco Medical AG, Switzerland) was used to analyze the tibial fracture ends of rats in each group. The fractured end of the left tibia was scanned with high resolution and 10 μm thickness. Each group of images was reconstructed in 3-D under the same conditions, and morphometric analysis was performed by the evaluation software of the μCT system. 3-D morphometric parameters include: trabecular relative volume (BV/TV), bone mineral density (BMC/TV), trabecular number (Tb.N), trabecular density (Tb.Th) and trabecular separation (Tb.Sp).

### Histological Analysis

Paraffin sections of tibial tissues were routinely prepared after decalcification of the tissues at the tibial fracture in rats. The paraffin sections were stained with hematoxylin and eosin (H&E) using an H&E kit (BL700A, Biosharp, China). In short, the sections were successively dehydrated in xylene and dewaxed in gradient ethanol, followed by staining with hematoxylin. The sections differentiated with 1% hydrochloric acid were counterstained with eosin. After dehydration and transparency treatment, the sections were sealed with neutral balsam (36313ES60, Yeasen, China), and the changes in tibial tissue were observed under the microscopy (BX53M, Olympus, Japan). The scoring standard of fracture healing in rats refers to the previous literature (Huo et al., 1991).

### Biomechanical Testing

Biomechanical testing was performed by a three-point bending test using a biomechanical testing machine (INSTRON3382, Instron, United States). Briefly, the tibia was placed on two fulcrums at a distance of 17 mm, and the load stress was loaded at the midpoint of the callus area. The loading bar was loaded at a rate of 1 mm/min until the tibia specimen was broken. The data were recorded and analyzed by the software of the mechanical experiment tester. The measured biomechanical parameters include ultimate load (N) and bending strength (MPa).

### Enzyme-Linked Immunosorbent Assay (ELISA)

The levels of vascular endothelial growth factor (VEGF), platelet-derived growth factors (PDGF), and basic fibroblast growth factor (bFGF) in rat serum were detected by ELISA kits (SBJ-R0130/SBJ-R0578/SBJ-R0817, SBJBIO, China). Briefly, rat serum was added to an antibody-coated 96-well plate followed by the addition of the HRP-labeled antibody. Subsequently, TMB was used for color development, and the optical density (OD) value was measured with a microplate reader (CMaxPlus, MD, China) at a wavelength of 450 nm, and the contents of VEGF, PDGF, and bFGF were calculated by the standard curve.

### Quantitative Reverse Transcription-Polymerase Chain Reaction (qRT-PCR)

Total RNA was extracted from the tibial callus tissue of rats by Total RNA Isolation Reagent (BS259A, Biosharp, China). After that, cDNA was synthesized through First Strand cDNA Synthesis Kit (abs601510, absin, China), which was then amplified in a Real-Time PCR System (7500, ThermoFisher, United States) with qPCR Mix (abs60086, absin, China). The amplification conditions of real-time PCR were as follows: 40 cycles of 95°C for 30 s, 60°C for 30 s, and 72°C for 30 s. Melting curves were obtained, and the sequences of primers used were designed, synthesized from Sangon Biotech Co., Ltd. (Shanghai, China), and summarized as follows: COLA1A, forward: 5′-GGA​GAG​AGC​ATG​ACC​GAT​GG-3′ and reverse: 5′-GGT​GGG​AGG​GAA​CCA​GAT​TG-3’; RUNX2, forward: 5′-CGC​CTC​ACA​AAC​AAC​CAC​AG-3′ and reverse: 5′-TCA​CTG​CAC​TGA​AGA​GGC​TG-3’; BMP2, forward: 5′-TGC​TTC​TTA​GAC​GGA​CTG​CG-3′ and reverse: 5′-GGG​GAA​GCA​GCA​ACA​CTA​GA-3’; Aggrecan, forward: 5′-AGC​CCT​TGT​CTG​AAT​GGA​GC-3′ and reverse: 5′-GTT​GGT​TTG​GAC​GCC​ACT​TC-3’; MMP13, forward: 5′-TCC​ATC​CCG​AGA​CCT​CAT​GT-3′ and reverse: 5′-CTC​AAA​GTG​AAC​CGC​AGC​AC-3’; GAPDH, forward: 5′-AGG​AAA​TGA​TGA​CCT​CCT​GAA​CT-3′ and reverse: 5′-GAA​GAT​GCG​GTC​ACC​TCA​CA -3’. The relative expressions of collagen type I alpha 1 (COL1A1), RUNX2, Bone morphogenetic protein 2 (BMP2), Aggrecan, and matrix metallopeptidase-13 (MMP13) were normalized to Glyceraldehyde-3-phosphate dehydrogenase (GAPDH) using the 2^−ΔΔCT^ approach.

### Serum Metabolomics Analysis

After 21°days of HPASD treatment, rats in the control group (sham), fracture-only group (frac), fracture + TBI group (FBI), and HPASD group (9.6 g/kg, FBIH) were anesthetized with 1% pentobarbital sodium, and blood was taken from abdominal aorta of rats. The rat serum was analyzed by UPLC-Q/TOF-MS/MS. The serum of rats in each group was analyzed by principal component analysis (PCA). The biomarkers were identified by the obtained accurate molecular weight and multi-stage mass spectrometry information combined with HMDB, Lipidomics Gateway, Metlin, and KEGG databases. MetaboAnalyst 3.0 was used to construct and analyze the metabolic pathway of the identified differential metabolites.

### Western Blot Analysis

Total protein was extracted from tissues by RIPA lysis buffer (R0010, Solarbio, China). And then, protein concentration quantification was performed by using a Bicinchoninic acid (BCA) Protein Assay kit (BI-WB005, SBJBIO, China). Afterwards, equal contents of protein (45 µg) were separated by SDS-PAGE and then transferred into a membrane that was blocked in 5% bovine serum albumin (BSA; BL-082, SBJBIO, China). Thereafter, the membrane was incubated with primary antibodies (4 °C, overnight) and secondary antibodies (room temperature, 1 h). The antibodies were purchased from Abcam (United States), including VEGFA (ab214424, 1/1000), COL1A1 (ab260043, 1/1000), RUNX2 (ab76956, 1/1000), BMP2 (ab284387, 1/1000), Aggrecan (ab3773, 1/1000), MMP13 (ab39012, 1/3000), PI3K p85 (ab191606, 1/1000), p-AKT Thr308 (ab38449, 1/1000), AKT (ab8805, 1/500), β-actin (ab8226, 1/1000), goat anti-rabbit (ab205718, 1/10,000) and goat anti-mouse (ab205719, 1/10,000). p-AKT Ser473 antibody (#AF8355, 1/2000) was obtained from Affinity Biosciences (Jiangsu, China). Visualization of protein expression was conducted by ECL kit (PE0010, Solarbio, China) on eZwest Lite Auto Imaging System (Genscript, United States).

### Statistical Analysis

Data were presented as mean ± standard deviation. One-way analysis of variance (ANOVA) was adopted for the comparison among the multiple groups, and a pairwise comparison between groups was conducted by the unpaired Student’s t-test. The statistical analysis was implemented with SPSS 16.0 software, with *p* < 0.05 considering statistical significance.

## Results

### Active Ingredients in HPASD

**FIGURE 2:**
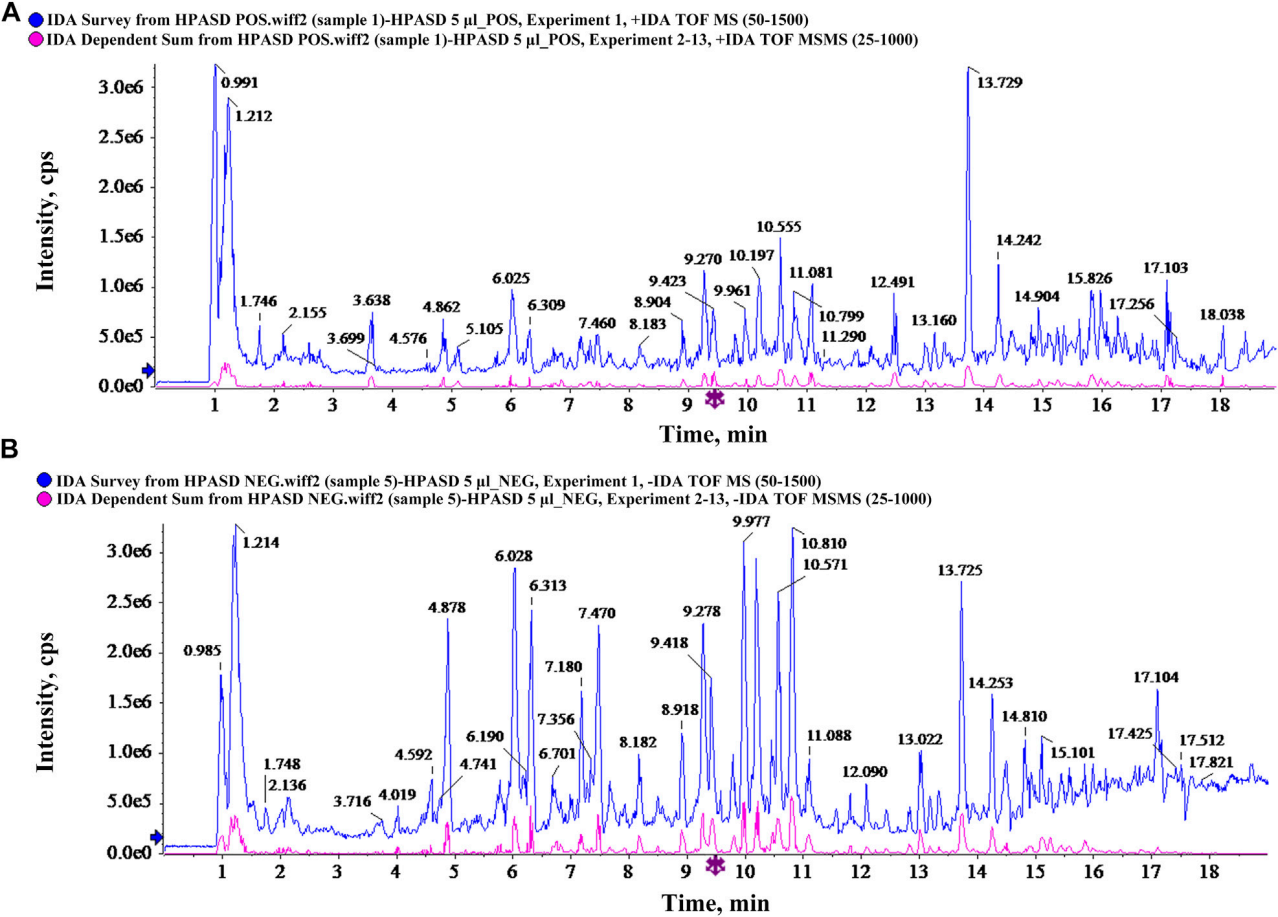
Characteristics of chemical components in HPASD by UPLC-Q-TOF/MS analysis. **(A)** Base peak chromatograms of HPASD in positive ion mode **(B)** Base peak chromatograms of HPASD in negative ion mode.

We analyzed the composition of HPASD using UPLC-Q/TOF-MS. Base peak chromatograms of HPASD in positive ion mode and negative ion mode were shown in Figures 2A, B. The compounds were qualitatively identified by comparison and screening with the TCM MS/MS Library database configured by SCIEX OS software. There were 94 attributed chromatographic peaks in the positive ion mode (Supplementary Table S1) and 68 attributed chromatographic peaks in the negative ion mode (Supplementary Table S2). Most of these compounds were flavonoids, and it can be conjectured that the major role to treat Fracture + TBI is flavonoids.

### HPASD Accelerated Fracture Healing in the Rat Model of TBI Combined With Tibial Fracture

**FIGURE 3:**
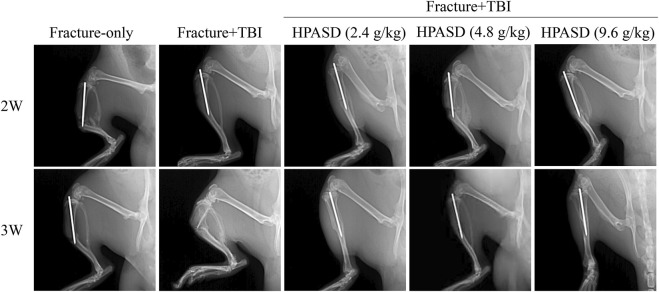
HPASD accelerated fracture healing in the rat model of traumatic brain injury. Representative X-ray images were used to show fracture healing in rats with traumatic brain injury (TBI) in the second and third weeks after HPASD treatment.

As shown in Figure 3, the fracture line in the fracture-only group was clear after 2°weeks, and no or little callus formation was found. At 3°weeks in the fracture-only group, the fracture line was still clear, with a small amount of callus forming. At 2°weeks in the fracture + TBI group, the fracture line was still clearly visible, with a small amount of callus forming and growing into the fracture end. At 3 weeks in the fracture + TBI group, the fracture line was blurred, and X-ray images showed that the fracture + TBI group had more faster compact bone substance formation than the fracture-only group. Significantly, HPASD blurred the fracture line in the fracture + TBI group, particularly in the high-dose (9.6 g/kg) and promoted callus formation and growth into the fracture end. However, there was no obvious significant healing in Fracture + TBI (HPASD 4.8 g/kg) group compared with the Fracture + TBI group only at 2 weeks. Interestingly, X-ray results also showed an obvious fracture-healing in Fracture + TBI (HPASD 4.8 g/kg) group after HPASD treatment for 3°weeks. The results indicated that the HPASD could be used as a therapeutic reagent for bone formation and bone healing in FBI rats to some extent.

### HPASD Promoted the Formulation of Fracture Callus in the Rat Model of TBI Combined With Tibial Fracture

**FIGURE 4:**
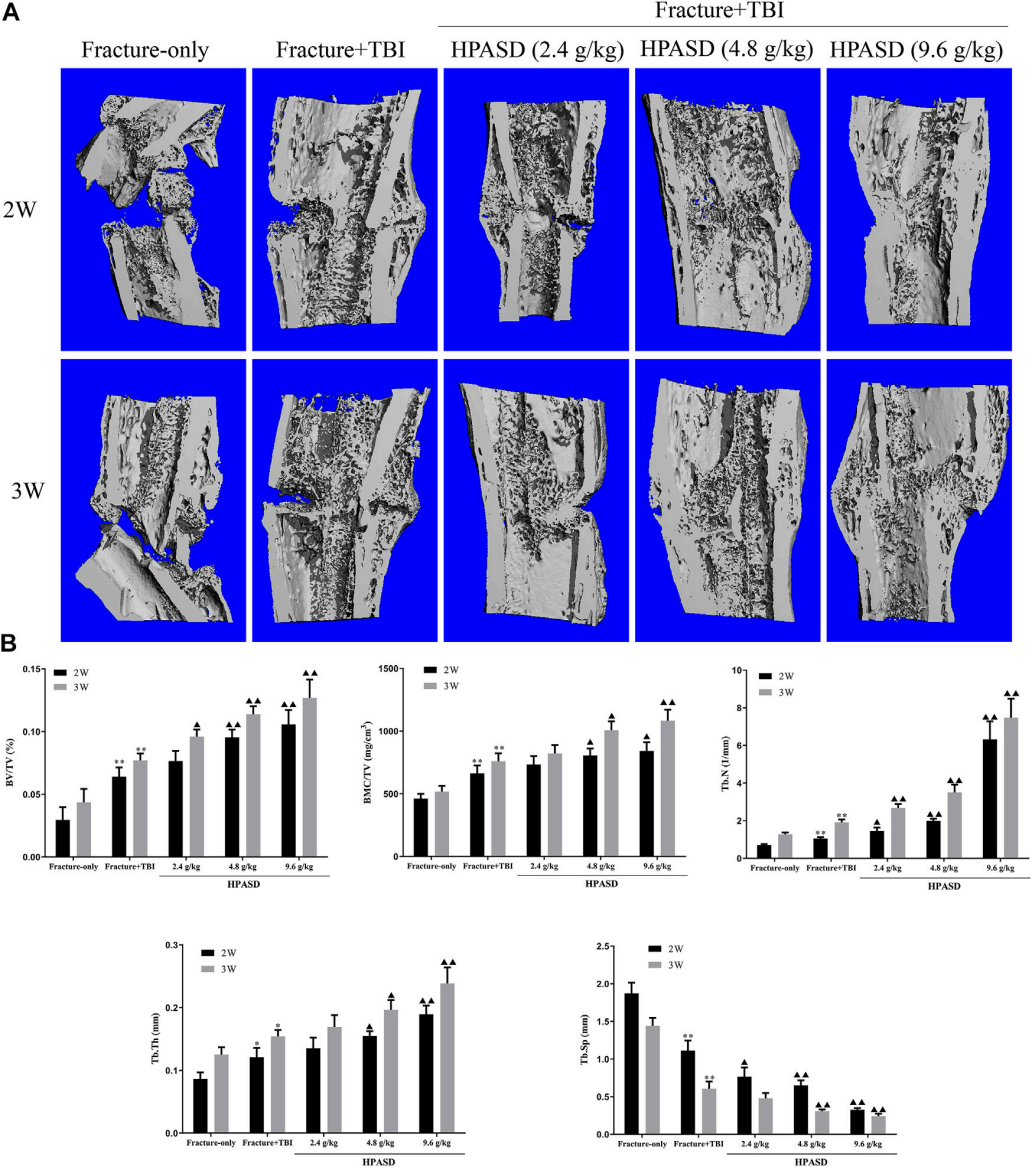
HPASD promoted the formulation of fracture callus in the rat model of traumatic brain injury. **(A)** Longitudinal cut view of Region Of Interest (ROI) in fracture site was displayed by Micro-CT analysis. **(B)** Quantitative analysis of BV/TV, BMC/TV, Tb. N, Tb. Tn, and Tb. Sp in ROI at the second and third weeks after HPASD treatment. Values are mean ± SD. ^*^ *p < 0.05*, ^**^ *p < 0.01* vs. Fracture-only group; ^▲^ *p < 0.05*, ^▲▲^ *p < 0.01* vs. Fracture + TBI group [*ANOVA*].

To further observe the protective effect of HPASD, the callus was evaluated by Micro-CT scanning post-operative day 14 and day 21 (Figure 4A). Similar to the X-ray findings, the HPASD groups formed significantly more callus tissue and observed more and denser trabecular structures on days 14 and 21 compared to the fracture + TBI group and showed higher mineralization compared to the fracture-only group. In addition, on the 14th and 21st days, BV/TV, BMC/TV, TB.N, and Tb.Th of the fracture + TBI group were higher than those of the fracture-only group but lower than those of HPASD groups, while Tb. Sp of the fracture + TBI was lower than that of the fracture-only group but higher than those of HPASD groups (Figure 4B, *p* < 0.05).

### HPASD Elevated the Histological Healing Scores in the Rat Model of TBI Combined With Tibial Fracture

**FIGURE 5:**
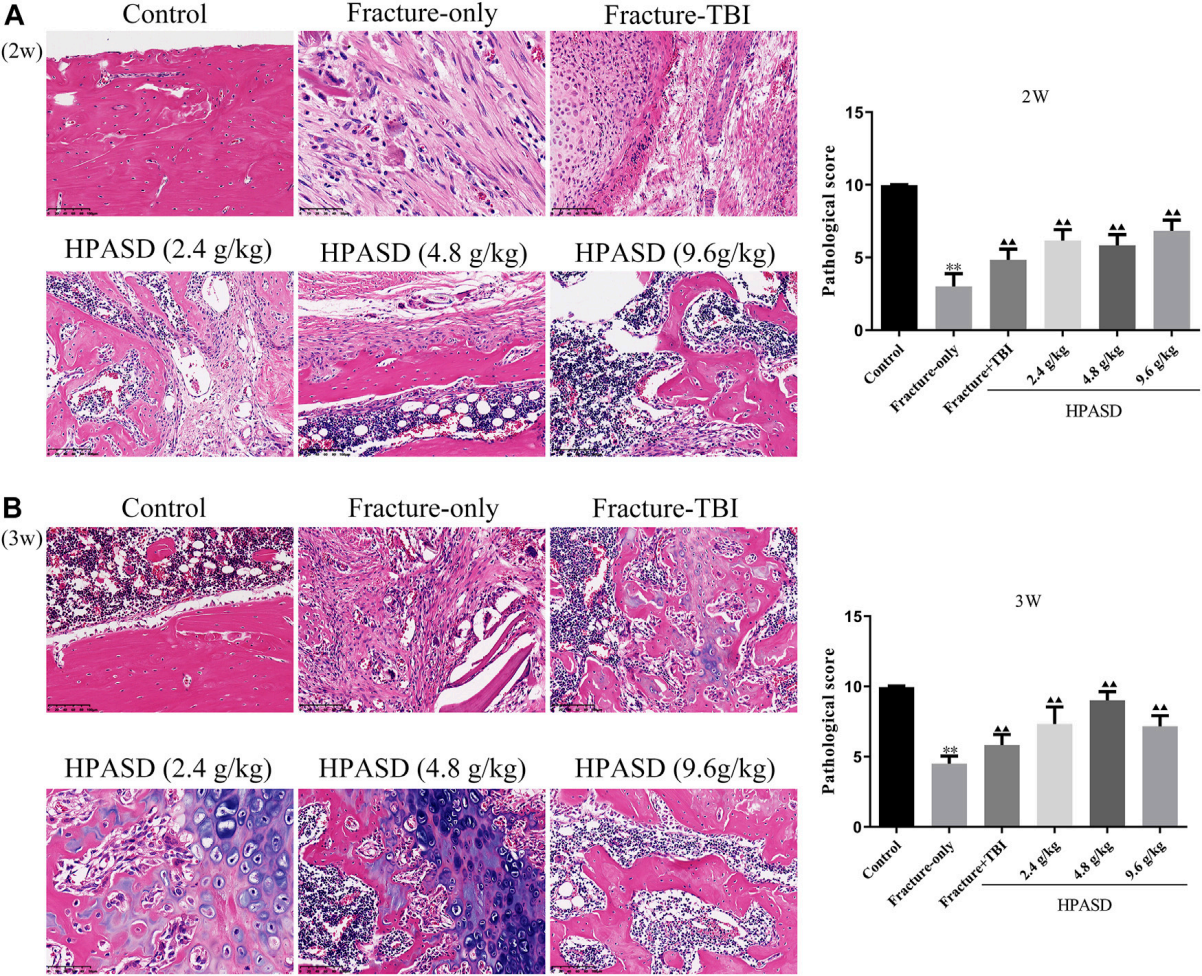
The histological analysis of the fracture healing in the rat model of traumatic brain injury. HE staining for evaluating healing of tibia shaft fracture at the **(A)** second and **(B)** third weeks after HPASD treatment (Scale bar: 100 μm). ^*^ *p < 0.05*, ^**^ *p < 0.01* vs. control group; ^▲^ *p < 0.05*, ^▲▲^ *p < 0.01* vs. Fracture-only group [unpaired Student’s t-test].

According to the results of H&E staining (Figures 5A, B), we found that in the fracture group, more fibrous tissue and cartilage tissue appeared on the 14th day, but no immature bone tissue was found. On the 21st day, fibrous tissue was replaced by bone tissue, and some mature bone tissue appeared, while in the fracture + TBI group, fibrous tissue was gradually replaced by bone tissue and some mature bone tissue appeared on 14th day and 21st day. In the HPASD group, more mature bone tissues appeared on both days 14 and 21. In addition, the histological score in the fracture + TBI group was higher than that in the fracture-only group but lower than that in the HPASD groups (Figures 4A, B, *p* < 0.01).

### HPASD Preserved Bone Strength in the Tibia of the Rat Model of TBI Combined With Tibial Fracture

**FIGURE 6:**
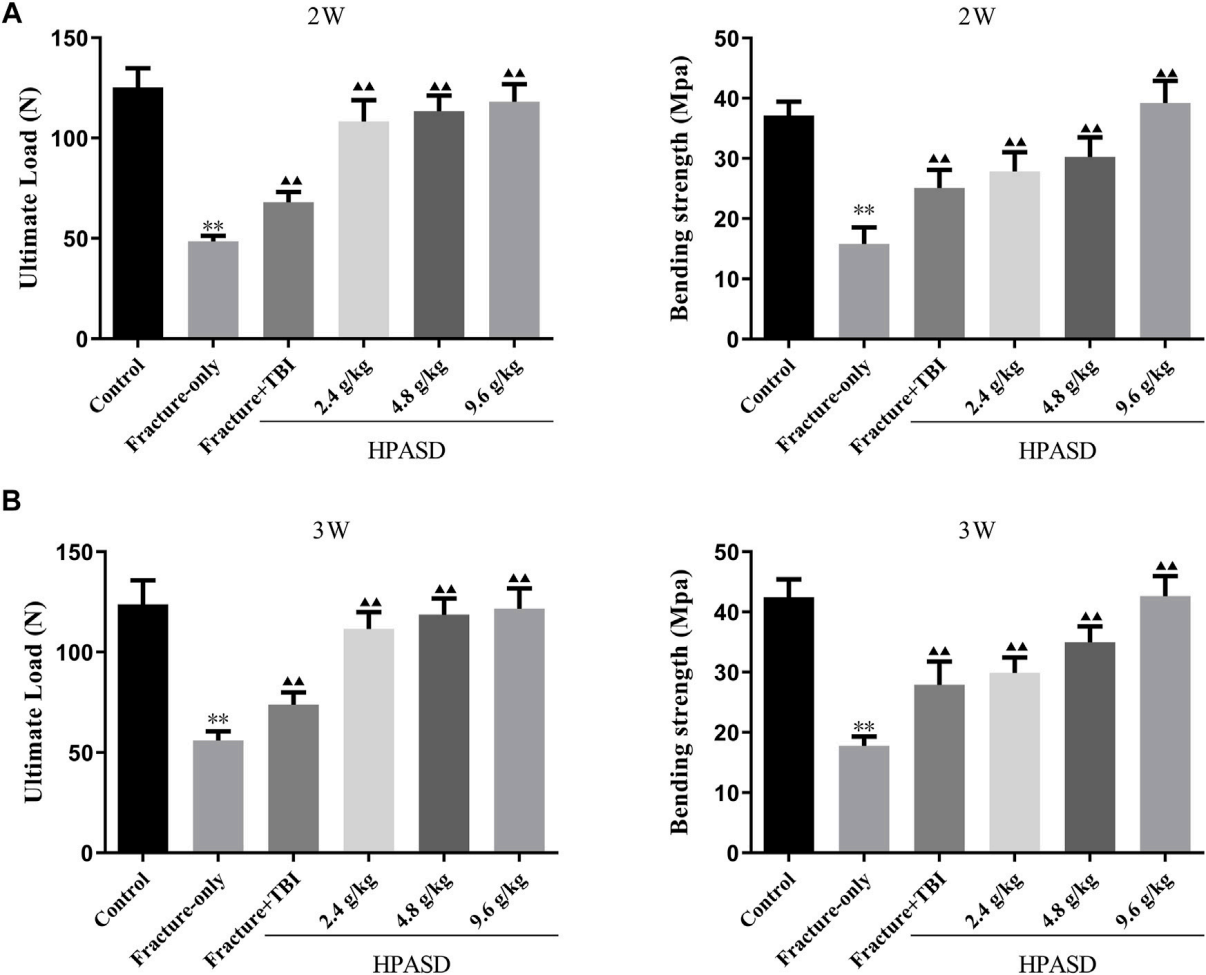
HPASD preserved bone strength in the tibia of TBI rats. The results of the three-point bending examination of **(A)** ultimate load and **(B)** bending strength in the tibia of TBI rats with HPASD treatment. ^*^ *p < 0.05*, ^**^ *p < 0.01* vs. Fracture-only group; ^▲^ *p < 0.05*, ^▲▲^ *p < 0.01* vs. Fracture + TBI group [unpaired Student’s t-test].

The ultimate load and bending strength of rats decreased significantly on days 14 and 21 after fracture, but those in the fracture + TBI group were slightly higher than those in the fracture-only group (Figures 6A, B, *p* < 0.01). However, HPASD facilitated the ultimate load and bending strength of fracture + TBI rats in a concentration-dependent manner (Figures 6A, B, *p* < 0.01).

### HPASD Facilitated the VEGF, PDGF, and bFGF Levels in the Rat Model of TBI Combined With Tibial Fracture

**FIGURE 7:**
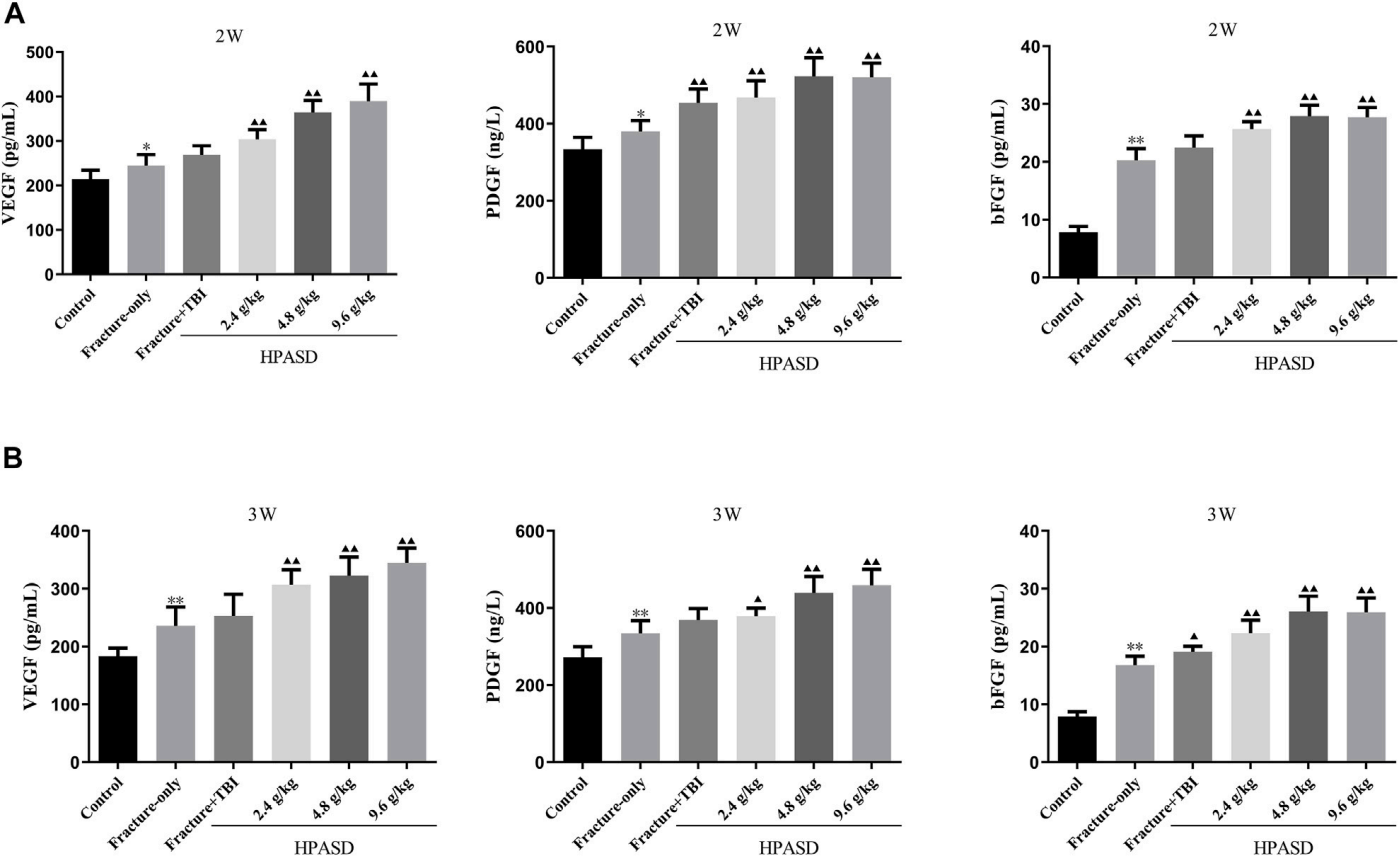
HPASD promoted the VEGF, PDGF, and bFGF levels in the rat model of traumatic brain injury. The concentrations of serum of VEGF, PDGF, and bFGF in the fracture rat model of traumatic brain injury at the **(A)** second and **(B)** third weeks after HPASD treatment were measured by ELISA assays, respectively. ^*^ *p < 0.05*, ^**^ *p < 0.01* vs. Fracture-only group; ^▲^ *p < 0.05*, ^▲▲^ *p < 0.01* vs. Fracture + TBI group [unpaired Student’s t-test].

Serum levels of VEGF, PDGF, and bFGF in rats were determined by ELISA kits and were elevated 14 and 21 days after the fracture. At the same time, the levels of VEGF, PDGF, and bFGF in the fracture + TBI group were higher than those in the fracture-only group on days 14 and 21 after modeling. Moreover, the levels of VEGF, PDGF, and bFGF in the fracture + TBI group were further increased after treatment with different concentrations of HPASD (Figures 7A, B, *p* < 0.05).

### Metabolic Changes in Rats Treated With HPASD

**FIGURE 8:**
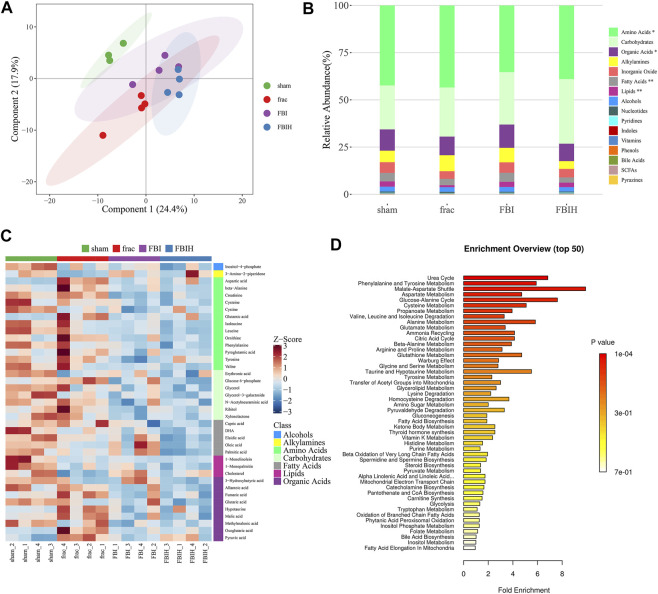
Aberrant metabolic patterns in serum samples in different groups. **(A)** PLS-DA score plot of serum samples urine samples discrimination of the metabolome between four groups, the control vs. Fracture-only vs. Fracture + TBI vs HPASD (9.6 g/kg) treated group. **(B)** Taxonomic distributions of metabolite in the different groups **(C)** Heatmap visualization of the differential metabolites identified in the serum samples from the different groups **(D)** Metabolic pathway analysis of the differential metabolites of the serum samples from the different groups.

We analyzed the metabolic characteristics of four groups of rats by OPLS-DA analysis. The score plot presented the clustering of sham, fracture, FBI and FBIH groups (Figure 8A). Figure 8B showed the changes in metabolite abundance of the four groups, with the changes in amino acids, organic acids, and lipids being significant. Then, we analyzed the expression differences of four groups of important metabolites and showed the most significant potential markers by heat map (Figure 8C). The top 50 pathways obtained by pathway enrichment analysis of differential metabolites were shown in Figure 8D.

### HPASD Promoted the Expressions of Osteogenic Factors in the Rat Model of TBI Combined With Tibial Fracture by Activating the PI3K/AKT Pathway

**FIGURE 9:**
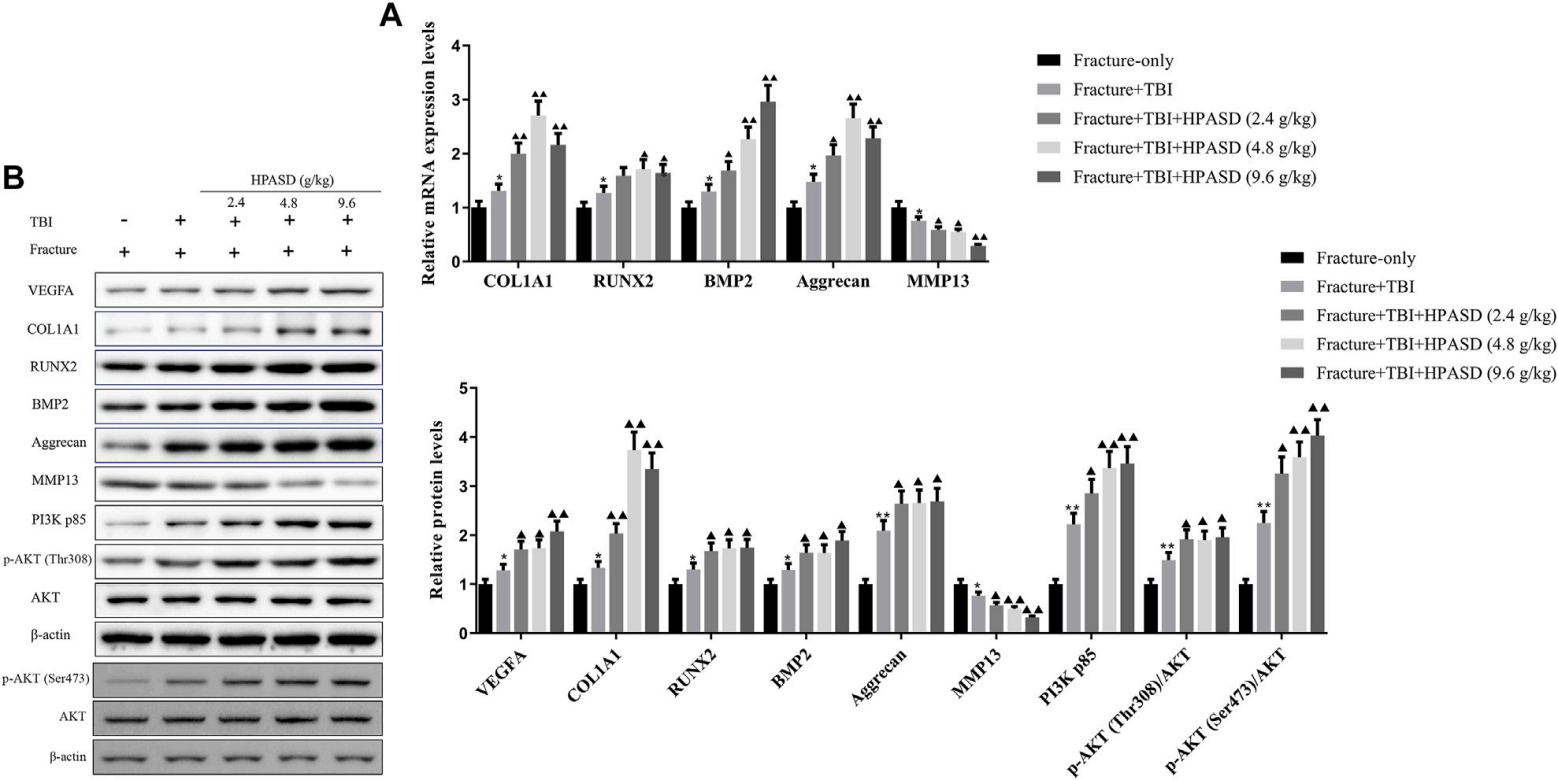
HPASD promoted the expressions of osteogenic factors in the rat model of traumatic brain injury by activating PI3K/AKT pathway. **(A)** The mRNA expression levels of osteogenic factors, COL1A1, RUNX2, BMP2, Aggrecan, and inflammation-related factor, MMP13 were evaluated by real-time PCR **(B)** Representative western blots and quantitative analysis of the VEGFA, COL1A1, RUNX2, BMP2, Aggrecan, MMP13, PI3K p85, p-AKT (Thr308), p-AKT (Ser473), and AKT in the callus tissues of TBI rats in each group. ^*^ *p < 0.05*, ^**^ *p < 0.01* vs. Fracture-only group; ^▲^ *p < 0.05*, ^▲▲^ *p < 0.01* vs. Fracture + TBI group [unpaired Student’s t-test].

Twenty-one days after administration of HPASD, total mRNA or protein was extracted from the callus tissue and analyzed by qRT-PCR and western blot. The results unveiled that the mRNA and protein levels of COL1A1, RUNX2, BMP2 and aggrecan in the fracture + TBI group were higher than those in the fracture-only group, while the treatment of HPASD further increased these mRNA levels in the fracture + TBI group (Figures 9A, B, *p* < 0.05). However, the level of MMP-13 in fracture + TBI group was lower than that in fracture-only group, which was further reduced by HPASD treatment. Furthermore, the levels of VEGFA, PI3K p85, p-AKT (Thr308)/AKT, and p-AKT (Ser473)/AKT in the fracture + TBI group were higher than those in the fracture-only group, which were further increased by HPASD (Figure 9B, *p* < 0.05).

## Discussion

Hu, Po Anshen decoction (HPASD), a combination of five herbal medicines, works synergistically to promote fracture healing. In this study, we identified the main active components of HPASD through UPLC/Q-TOF MS analysis combined with database retrieval. Among these active components, Cordycepin (Li et al., 2020), Chlorogenic acid (Liu L. et al., 2018), Puerarin (Wong and Rabie, 2007), Hyperin (Saul et al., 2021) and Naringenin (Estai et al., 2011) have been proved to promote fracture healing. Meanwhile, these active ingredients can also improve TBI (Wang et al., 2014; Deng et al., 2021; Wei et al., 2021). These results present a pharmacological basis for HPASD in the treatment of TBI combined with fracture.

Bone mineral density (BMD) is the main predictor of fracture healing, and bone microstructure is an important factor affecting bone strength. In this study, we observed bone healing by X-ray and bone microstructure by micro CT. Our results showed that TBI can promote callus formation, increase bone density and improve the bone microstructure around the fracture, which were consistent with previous reports (Han et al., 2021). In addition, we also found that HPASD accelerated fracture healing in a concentration-dependent manner, which was also supported by the results of bone mechanical properties test and histological staining. Although there is no report of HPASD on accelerating fracture healing, the active ingredients in HPASD, such as puerarin, have been proved to have the effects of increasing bone density and improving trabecular bone structure (Yang et al., 2018), and cordycepin can improve the mechanical properties of bone and promote the osteogenesis of bone marrow mesenchymal stem cells (Li et al., 2020). Therefore, the promoting effect of HPASD on fracture healing is the result of the synergistic effect of various active ingredients.

It is reported that the mechanism of TBI accelerating fracture healing may be related to the special function of cytokines (Mollahosseini et al., 2021). PDGF is one of the bone growth factors, which can promote bone formation and reconstruction (Peng et al., 2020). BFGF is a mitogenic growth factor, which can accelerate the repair of bone tissue (Zhang et al., 2017). VEGF is essential for neovascularization after injury, and angiogenesis can participate in the recovery of blood supply during osteogenesis. Importantly, PDGF, VEGF and bFGF can synergistically promote the prognosis of fracture (Cao et al., 2003). It has been proved that the expression of these three cytokines in fracture tissues is involved in the mechanism of accelerating fracture healing in TBI patients (Zhang et al., 2018; Mollahosseini et al., 2021). Similarly, we found that the serum of TBI rats with fracture secreted more PDGF, VEGF and bFGF than that of rats with fracture alone, and HPASD could further promote the secretion of these three cytokines, suggesting that HPASD could accelerate angiogenesis and fracture healing by regulating the secretion of cytokines.

Mesenchymal stem cells (MSC) exert an important effect on bone formation and differentiation (Garg et al., 2017). RUNX2 is a key transcription factor that regulates MSC differentiation into osteoblasts, and BMP2 is involved in stimulating MSC differentiation into osteoblasts (Garg et al., 2017). Aggrecan and COL1A1 are key proteins in cartilage formation, while MMP13 can degrade the extracellular matrix which is important for osteoblast growth and differentiation, leading to bone defects (Komori, 2018). Interestingly, cordycepin in the active components of HPASD can induce the expression of BMP2 and RUNX2, Naringenin can up-regulate COL1A1 and Aggrecan (Devraj et al., 2019), and Chlorogenic acid can inhibit MMP13 (Liu et al., 2017). Consistent with previous studies, we found that HPASD can increase the expression of BMP2, RUNX2, COL1A1 and aggrecan, and inhibit the level of MMP13, indicating that HPASD can accelerate fracture healing by promoting bone formation and differentiation of MSC.

The injury and healing of fracture are related to the activation of the PI3K/AKT signal pathway. Activating PI3K/AKT pathway can not only promote the osteogenic differentiation of bone marrow stem cells (Zhao et al., 2020) but also enhance the viability of osteoblasts (Xie et al., 2020). In addition, the activation of the PI3K/AKT pathway is one of the mechanisms that the fracture healing rate of TBI patients is faster than that of single fracture patients (Hu et al., 2021). Chlorogenic acid, the active ingredient of HPASD, has been reported to increase femoral bone mineral density and promote osteogenic differentiation of bone marrow stem cells by activating PI3K/AKT pathway (Zhou et al., 2016), and Puerarin was proved to stimulate the human osteoblast differentiation and prevent osteoporosis by activating the PI3K/AKT (Wang et al., 2013). Similarly, we found that HPASD can enhance the activation of PI3K/AKT induced by TBI, indicating that HPASD may accelerate fracture healing by activating PI3K/AKT pathway.

Our serum metabolomics analysis showed that the levels of lipid, amino acids and, organic acid in HPASD treatment or TBI combined with the fracture group were different from those in fracture group alone, and the abnormalities of these metabolites have been reported to be related to orthopedic problems (Wijnands et al., 2012; Wang et al., 2013; Ghorabi et al., 2019). In addition, these metabolites are more abundant in malate-aspartate shuttle and glucose-alanine cycle, which are necessary for the glycolysis process required for osteoblast differentiation (Lee et al., 2020). Therefore, the mechanism of HPASD accelerating fracture healing may involve aerobic glycolysis of osteoblasts to influence osteoblast differentiation. However, the current study has various shortcomings. Firstly, the pharmacological effects of active ingredients in HPASD have not been determined in this study. In addition, it is worth testing the roles of differential metabolites in the progress of HPASD-treated fracture combined with traumatic brain injury. Moreover, additional signaling pathways may also be associated with the accelerated fracture-healing effect of HPASD. Therefore, further basic studies and clinical experiments are required to assess the molecular mechanism of HPASD in the treatment of fracture combined with traumatic brain injury *in vitro* and *in vivo*.

In summary, these results illustrated the pharmacological mechanism of HPASD in the treatment of fracture combined with TBI via promoting bone formation to accelerate fracture healing, specifically by activating the PI3K/AKT pathway.

## Data Availability

The original contributions presented in the study are included in the article/Supplementary Material further inquiries can be directed to the corresponding authors.
