# Supplementary material for: Hu’po Anshen Decoction Accelerated Fracture-Healing in a Rat Model of Traumatic Brain Injury Through Activation of PI3K/AKT Pathway
Source: Front Pharmacol. 2022 Jul 18;13:952696. doi: 10.3389/fphar.2022.952696 (PMC9341486; doi:10.3389/fphar.2022.952696)
Supplement: Supplementary file 1 [file Table1.DOC]

**Table S1** **Compounds identified of HPASD in positive mode**

| No. | Component Name | Adduct | Area | Retention Time | Formula | Precursor Mass | Found At Mass | Mass Error (ppm) | Library Score |
| --- | --- | --- | --- | --- | --- | --- | --- | --- | --- |
| 1 | L(+)-Arginine | [M+H] | 880300 | 1.11 | C6H14N4O2 | 175.119 | 175.1189 | -0.3 | 100 |
| 2 | 1-Deoxynojirimycin | [M+H] | 104200 | 1.12 | C6H13NO4 | 164.092 | 164.092 | 1.5 | 79.5 |
| 3 | Glutamic acid | [M+H] | 118800 | 1.15 | C5H9NO4 | 148.06 | 148.0608 | 2.5 | 97.2 |
| 4 | Betaine | [M+H] | 587700 | 1.16 | C5H11NO2 | 118.086 | 118.0864 | 0.9 | 100 |
| 5 | Trigonelline | [M+H] | 1246000 | 1.2 | C7H7NO2 | 138.055 | 138.0546 | -2.4 | 93.8 |
| 6 | Proline | [M+H] | 2208000 | 1.22 | C5H9NO2 | 116.071 | 116.0705 | -1.3 | 98.1 |
| 7 | Stachydrine | [M+H] | 827000 | 1.24 | C7H13NO2 | 144.102 | 144.1018 | -0.5 | 99 |
| 8 | Adenine | [M+H] | 490800 | 1.24 | C5H5N5 | 136.062 | 136.0618 | 0.2 | 83.6 |
| 9 | Nicotinic acid | [M+H] | 177400 | 1.75 | C6H5NO2 | 124.039 | 124.0394 | 0.7 | 97.8 |
| 10 | Nicotinamide | [M+H] | 168400 | 1.83 | C6H6N2O | 123.055 | 123.0551 | -1.3 | 96 |
| 11 | 6-Hydroxypurine | [M+H] | 24460 | 1.91 | C5H4N4O | 137.046 | 137.046 | 1.2 | 88.4 |
| 12 | Leucine | [M+H] | 584200 | 2.49 | C6H13NO2 | 132.102 | 132.1019 | 0 | 87.1 |
| 13 | Adenosine | [M+H] | 769500 | 2.59 | C10H13N5O4 | 268.104 | 268.1041 | 0.2 | 98 |
| 14 | Cordycepin | [M+H] | 62280 | 2.68 | C10H13N5O3 | 252.109 | 252.1093 | 0.7 | 98.5 |
| 15 | Guanosine | [M+H] | 104100 | 2.77 | C10H13N5O5 | 284.099 | 284.0992 | 0.9 | 99.1 |
| 16 | Protocatechuic acid | [M+H] | 20800 | 3.58 | C15H24N2O | 249.196 | 249.1959 | -0.9 | 92.9 |
| 17 | Phenylalanine | [M+H] | 759900 | 3.64 | C9H11NO2 | 166.086 | 166.0862 | -0.3 | 99 |
| 18 | Harpagide | [M+H] | 23240 | 4.17 | C15H24O10 | 382.171 | 382.1711 | 1 | 91 |
| 19 | Ephedrine | [M+H] | 55680 | 5.03 | C10H15NO | 166.123 | 166.1228 | 0.7 | 98.6 |
| 20 | Esculin hydrate | [M+H] | 40240 | 5.33 | C15H16O9 | 341.087 | 341.0871 | 1 | 95.8 |
| 21 | Protocatechuic Aldehyde | [M+H] | 45910 | 5.61 | C7H6O3 | 139.039 | 139.039 | 0 | 99.1 |
| 22 | Mulberroside A | [M+H] | 5961 | 5.67 | C26H32O14 | 569.186 | 569.1864 | -0.1 | 95.7 |
| 23 | Chlorogenic acid | [M+H] | 1364000 | 6.02 | C16H18O9 | 355.102 | 355.1023 | -0.2 | 99.4 |
| 24 | Daphnetin | [M+H] | 72920 | 6.53 | C9H6O4 | 179.034 | 179.0337 | -0.8 | 92.1 |
| 25 | Puerarin | [M+H] | 181800 | 6.91 | C21H20O9 | 417.118 | 417.1184 | 0.9 | 93.7 |
| 26 | Geniposide | [M+H] | 21120 | 7.18 | C17H24O10 | 406.171 | 406.1713 | 1.2 | 97 |
| 27 | Vitamin B2 | [M+H] | 60150 | 7.2 | C17H20N4O6 | 377.146 | 377.1459 | 0.9 | 94.8 |
| 28 | Albiflorin | [M+H] | 668500 | 7.66 | C23H28O11 | 481.17 | 481.1707 | 0.6 | 91.1 |
| 29 | Pinoresinol Diglucoside | [M+H] | 5731 | 7.8 | C32H42O16 | 700.281 | 700.282 | 1.2 | 92.4 |
| 30 | Paeoniflorin | [M+H] | 298100 | 8.18 | C23H28O11 | 498.197 | 498.1974 | 0.8 | 95.5 |
| 31 | Prim-O-glucosylcimifugin | [M+H] | 113500 | 8.25 | C22H28O11 | 469.17 | 469.1709 | 1 | 93.9 |
| 32 | Eleutheroside E | [M+H] | 5933 | 8.37 | C34H46O18 | 760.302 | 760.3026 | 0.5 | 76.4 |
| 33 | Complanatoside | [M+H] | 10860 | 8.86 | C28H32O16 | 625.176 | 625.1771 | 1.3 | 99.3 |
| 34 | Rutin | [M+H] | 1203000 | 8.91 | C27H30O16 | 611.161 | 611.1606 | -0.1 | 97.2 |
| 35 | Isoscopoletin | [M+H] | 155600 | 8.96 | C10H8O4 | 193.05 | 193.0497 | 0.8 | 97.8 |
| 36 | Scopoletin | [M+H] | 155600 | 8.96 | C10H8O4 | 193.05 | 193.0497 | 0.8 | 91.5 |
| 37 | Isoferulic acid | [M+H] | 6514 | 9.09 | C10H10O4 | 195.065 | 195.0654 | 1 | 99.3 |
| 38 | IsoActeoside | [M+H] | 2754 | 9.22 | C29H36O15 | 642.239 | 642.2385 | -1.1 | 91.4 |
| 39 | Isoliquiritigenin | [M+H] | 150800 | 9.22 | C15H12O4 | 257.081 | 257.0808 | 0 | 96.6 |
| 40 | Quercetin | [M+H] | 1875000 | 9.27 | C15H10O7 | 303.05 | 303.0497 | -0.8 | 81.5 |
| 41 | Hyperin | [M+H] | 2110000 | 9.27 | C21H20O12 | 465.103 | 465.1026 | -0.4 | 100 |
| 42 | Luteoloside | [M+H] | 2433000 | 9.42 | C21H20O11 | 449.108 | 449.1077 | -0.3 | 100 |
| 43 | Luteolin-7-O-β-D-glucuronide | [M+H] | 184800 | 9.46 | C21H18O12 | 463.087 | 463.0876 | 1.1 | 99.5 |
| 44 | Cimifugin | [M+H] | 105500 | 9.67 | C16H18O6 | 307.118 | 307.1174 | -0.7 | 93.1 |
| 45 | Nodakenin | [M+H] | 499500 | 9.77 | C20H24O9 | 409.149 | 409.1494 | 0.3 | 93.2 |
| 46 | Aempferol-3-O-rutinoside | [M+H] | 319100 | 9.81 | C27H30O15 | 595.166 | 595.1657 | -0.2 | 97.6 |
| 47 | Luteolin | [M+H] | 1195000 | 10.19 | C15H10O6 | 287.055 | 287.0551 | 0.4 | 98.1 |
| 48 | 4'-O-β-Glucopyranosyl-5-O-methylvisamminol | [M+H] | 265500 | 10.3 | C22H28O10 | 453.176 | 453.1756 | 0.2 | 97.9 |
| 49 | Protopine | [M+H] | 409700 | 10.3 | C20H19NO5 | 354.134 | 354.1336 | 0.1 | 93.7 |
| 50 | Rhoifolin | [M+H] | 70280 | 10.41 | C27H30O14 | 579.171 | 579.1715 | 1.1 | 92.7 |
| 51 | Naringenin | [M+H] | 350300 | 10.46 | C15H12O5 | 273.076 | 273.0759 | 0.7 | 98.9 |
| 52 | Narirutin | [M+H] | 370000 | 10.46 | C27H32O14 | 581.186 | 581.1868 | 0.5 | 73.2 |
| 53 | Apigenin-7-glucoside | [M+H] | 3912000 | 10.56 | C21H20O10 | 433.113 | 433.1129 | -0.2 | 99.3 |
| 54 | Hesperidin | [M+H] | 75340 | 10.78 | C28H34O15 | 611.197 | 611.1977 | 1.1 | 86.6 |
| 55 | Dicaffeoylquinic Acid (Cynarin) | [M+H] | 848700 | 10.79 | C25H24O12 | 517.134 | 517.1341 | 0.2 | 98.9 |
| 56 | Pratensein-7-O-glucoside | [M+H] | 2436000 | 11.09 | C22H22O11 | 463.123 | 463.1229 | -1.3 | 94 |
| 57 | Isocorynoxeine | [M+H] | 167900 | 11.39 | C22H26N2O4 | 383.197 | 383.1967 | 0.3 | 85.7 |
| 58 | Benzoylmesaconine | [M+H] | 80410 | 11.41 | C31H43NO10 | 590.296 | 590.2966 | 1 | 95.3 |
| 59 | Canadine | [M+H] | 25200 | 11.66 | C20H21NO4 | 340.154 | 340.1546 | 0.6 | 75.2 |
| 60 | Corydaline | [M+H] | 143700 | 11.95 | C22H27NO4 | 370.201 | 370.201 | -0.7 | 96.8 |
| 61 | (±)-Isorhynchophylline | [M+H] | 192600 | 12.03 | C22H28N2O4 | 385.212 | 385.212 | -0.5 | 82.5 |
| 62 | Oxypeucedanin | [M+H] | 23930 | 12.09 | C16H14O5 | 287.091 | 287.0917 | 1 | 78.8 |
| 63 | 7-O-beta-D-glucuronide | [M+H] | 539400 | 12.09 | C21H18O11 | 447.092 | 447.0925 | 0.7 | 100 |
| 64 | Ononin | [M+H] | 160700 | 12.13 | C22H22O9 | 431.134 | 431.1339 | 0.6 | 98.6 |
| 65 | Nuciferin | [M+H] | 62910 | 12.18 | C19H21NO2 | 296.165 | 296.1644 | -0.5 | 97.9 |
| 66 | Daidzein | [M+H] | 104000 | 12.32 | C15H10O4 | 255.065 | 255.065 | -0.8 | 95.2 |
| 67 | Berberine | [M+H] | 2559000 | 12.49 | C20H17NO4 | 336.123 | 336.1226 | -1.3 | 98.5 |
| 68 | Benzoylhypacoitine | [M+H] | 52180 | 12.75 | C31H43NO9 | 574.301 | 574.3013 | 0.3 | 100 |
| 69 | Eriodictyol | [M+H] | 390900 | 12.83 | C15H12O6 | 289.071 | 289.0704 | -0.9 | 97.9 |
| 70 | Linarin | [M+H] | 49710 | 13.01 | C28H32O14 | 593.186 | 593.1863 | -0.3 | 98.3 |
| 71 | Camphor | [M+H] | 64470 | 13.02 | C10H16O | 153.127 | 153.1274 | 0.1 | 83.5 |
| 72 | Hamaudol Glycoside | [M+H] | 10100 | 13.32 | C21H26O10 | 439.16 | 439.1603 | 1 | 90.4 |
| 73 | Calycosin-7-O-glucoside | [M+H] | 9038000 | 13.73 | C22H22O10 | 447.129 | 447.1282 | -0.8 | 98.2 |
| 74 | Wogonin 7-O-glucuronide | [M+H] | 579200 | 13.82 | C22H20O11 | 461.108 | 461.1078 | -0.1 | 88.4 |
| 75 | Psoralen | [M+H] | 49030 | 14.14 | C11H6O3 | 187.039 | 187.0389 | -0.4 | 97.6 |
| 76 | Benzoylpaeoniflorin | [M+H] | 43930 | 14.19 | C30H32O12 | 602.223 | 602.2241 | 1.4 | 85 |
| 77 | Apigenin | [M+H] | 2480000 | 14.24 | C15H10O5 | 271.06 | 271.0594 | -2.6 | 99.2 |
| 78 | Hydroxygenkwanin | [M+H] | 291600 | 14.47 | C16H12O6 | 301.071 | 301.0705 | -0.4 | 84.2 |
| 79 | Paeonol | [M+H] | 311000 | 14.86 | C9H10O3 | 167.07 | 167.0697 | -3.5 | 98.9 |
| 80 | Bergapten | [M+H] | 108200 | 15.14 | C12H8O4 | 217.05 | 217.0494 | -0.8 | 86.2 |
| 81 | Isopimpinellin | [M+H] | 38880 | 15.16 | C13H10O5 | 247.06 | 247.0603 | 0.7 | 84.1 |
| 82 | Formononetin | [M+H] | 326400 | 15.17 | C16H12O4 | 269.081 | 269.0807 | -0.6 | 85 |
| 83 | Dictamnine | [M+H] | 75780 | 15.21 | C12H9NO2 | 200.071 | 200.0707 | 0.4 | 95.8 |
| 84 | Aurantio-Obtusin | [M+H] | 915900 | 15.26 | C17H14O7 | 331.081 | 331.081 | -0.7 | 89.1 |
| 85 | Irisflorentin | [M+H] | 35220 | 15.64 | C20H18O8 | 387.107 | 387.1078 | 0.8 | 87.7 |
| 86 | Chrysosplenetin B | [M+H] | 644600 | 15.77 | C19H18O8 | 375.107 | 375.1072 | -0.7 | 81.3 |
| 87 | Nobiletin | [M+H] | 1764000 | 15.82 | C21H22O8 | 403.139 | 403.1384 | -1 | 92.4 |
| 88 | Acacetin | [M+H] | 1108000 | 15.85 | C16H12O5 | 285.076 | 285.0756 | -0.5 | 99.3 |
| 89 | 3-N-butyl-4,5-dihydrophthalide | [M+H] | 246400 | 16.33 | C12H16O2 | 193.122 | 193.122 | -1.4 | 94 |
| 90 | Curcumol | [M+H] | 172500 | 16.86 | C15H24O2 | 237.185 | 237.1847 | -1 | 91.6 |
| 91 | Ligustilide | [M+H] | 488900 | 16.94 | C12H14O2 | 191.107 | 191.1063 | -1.9 | 92.2 |
| 92 | Pogostone | [M+H] | 15930 | 17.22 | C12H16O4 | 225.112 | 225.1124 | 1 | 77.1 |
| 93 | Alantolactone | [M+H] | 75620 | 17.23 | C15H20O2 | 233.154 | 233.1536 | -0.2 | 88.1 |
| 94 | Levistilide A | [M+H] | 22320 | 17.95 | C24H28O4 | 398.233 | 398.2327 | 0.2 | 72.8 |
