# Supplementary material for: Hu’po Anshen Decoction Accelerated Fracture-Healing in a Rat Model of Traumatic Brain Injury Through Activation of PI3K/AKT Pathway
Source: Front Pharmacol. 2022 Jul 18;13:952696. doi: 10.3389/fphar.2022.952696 (PMC9341486; doi:10.3389/fphar.2022.952696)
Supplement: Supplementary file 2 [file Table2.DOC]

**Table S2** **Compounds identified of HPASD in negative mode**

| No. | Component Name | Adduct | Area | Retention Time | Formula | Precursor Mass | Found At Mass | Mass Error (ppm) | Library Score |
| --- | --- | --- | --- | --- | --- | --- | --- | --- | --- |
| 1 | Glutamic acid | [M-H] | 17620 | 1.13 | C5H9NO4 | 146.046 | 146.0458 | -0.9 | 95.8 |
| 2 | D-(+)-Mannose | [M-H] | 761500 | 1.19 | C6H12O6 | 179.056 | 179.0563 | 0.8 | 82.7 |
| 3 | Quinic acid | [M-H] | 1889000 | 1.22 | C7H12O6 | 191.056 | 191.0562 | 0.5 | 89.8 |
| 4 | Maleic acid | [M-H] | 431200 | 1.28 | C4H4O4 | 115.004 | 115.0039 | 2 | 89 |
| 5 | Citric acid | [M-H] | 3029000 | 1.35 | C6H8O7 | 191.02 | 191.02 | 1.2 | 98.6 |
| 6 | Amber Acid | [M-H] | 104600 | 2.41 | C4H6O4 | 117.019 | 117.0194 | 0.8 | 92.1 |
| 7 | Adenosine | [M-H] | 10110 | 2.59 | C10H13N5O4 | 266.089 | 266.0902 | 2.6 | 76.8 |
| 8 | Guanosine | [M-H] | 155600 | 2.77 | C10H13N5O5 | 282.084 | 282.0846 | 0.7 | 97.6 |
| 9 | Gallic acid | [M-H] | 283100 | 2.88 | C7H6O5 | 169.014 | 169.0143 | 0.5 | 80.5 |
| 10 | Cinnamic acid | [M-H] | 33790 | 3.65 | C9H8O2 | 147.045 | 147.0452 | 0.3 | 93.4 |
| 11 | Phenylalanine | [M-H] | 245800 | 3.66 | C9H11NO2 | 164.072 | 164.0717 | 0.3 | 95.9 |
| 12 | Geniposidic acid | [M-H] | 42590 | 4.28 | C16H22O10 | 373.114 | 373.114 | -0.1 | 87 |
| 13 | Neochlorogenic acid | [M-H] | 9066000 | 4.88 | C16H18O9 | 353.088 | 353.088 | 0.6 | 98.1 |
| 14 | L-Tryptophan | [M-H] | 239300 | 5.13 | C11H12N2O2 | 203.083 | 203.0827 | 0.5 | 94.2 |
| 15 | Esculin hydrate | [M-H] | 178400 | 5.34 | C15H16O9 | 339.072 | 339.0725 | 1 | 72.1 |
| 16 | Vitamin B2 | [M-H] | 14550 | 5.45 | C17H20N4O6 | 375.131 | 375.1299 | -2.9 | 89.7 |
| 17 | Protocatechuic Aldehyde | [M-H] | 576700 | 5.61 | C7H6O3 | 137.024 | 137.0245 | 0.3 | 94 |
| 18 | Mulberroside A | [M-H] | 58320 | 5.68 | C26H32O14 | 567.172 | 567.1719 | 0 | 91 |
| 19 | Esculetin | [M-H] | 309500 | 6.53 | C9H6O4 | 177.019 | 177.0192 | -0.8 | 92.5 |
| 20 | Caffeic acid | [M-H] | 1234000 | 6.69 | C9H8O4 | 179.035 | 179.0351 | 0.7 | 92.2 |
| 21 | Puerarin | [M-H] | 322600 | 6.9 | C21H20O9 | 415.103 | 415.1031 | -1 | 96.3 |
| 22 | Pinoresinol Diglucoside | [M-H] | 16600 | 7.8 | C32H42O16 | 727.245 | 727.2445 | -1.3 | 90.8 |
| 23 | Schaftoside | [M-H] | 56450 | 7.94 | C26H28O14 | 563.141 | 563.1402 | -0.7 | 91.5 |
| 24 | Paeoniflorin | [M-H] | 2209000 | 8.18 | C23H28O11 | 525.161 | 525.1612 | -0.3 | 99.1 |
| 25 | p-Coumaric acid | [M-H] | 36010 | 8.31 | C9H8O3 | 163.04 | 163.04 | -0.4 | 90.9 |
| 26 | Eleutheroside E | [M-H] | 22590 | 8.37 | C34H46O18 | 787.267 | 787.2655 | -1.5 | 100 |
| 27 | 7-Hydroxycoumarin | [M-H] | 217200 | 8.81 | C9H6O3 | 161.024 | 161.0243 | -0.5 | 96.4 |
| 28 | Ellagic Acid | [M-H] | 175300 | 8.82 | C14H6O8 | 300.999 | 300.999 | -0.1 | 82.3 |
| 29 | Cichoric acid | [M-H] | 29560 | 8.86 | C22H18O12 | 473.073 | 473.0721 | -0.9 | 88.1 |
| 30 | Rutin | [M-H] | 3300000 | 8.92 | C27H30O16 | 609.146 | 609.1456 | -0.8 | 98.2 |
| 31 | IsoActeoside | [M-H] | 79480 | 9.22 | C29H36O15 | 623.198 | 623.1975 | -1 | 98.2 |
| 32 | Liquiritin | [M-H] | 308400 | 9.23 | C21H22O9 | 417.119 | 417.1186 | -1.1 | 97.2 |
| 33 | 2,3,5,4'-Tetrahydroxystilbene-2-O-b-D-glucoside | [M-H] | 62070 | 9.27 | C20H22O9 | 405.119 | 405.1188 | -0.7 | 70.6 |
| 34 | Hyperin | [M-H] | 8205000 | 9.28 | C21H20O12 | 463.088 | 463.0878 | -1 | 100 |
| 35 | Luteolin-7-O-β-D-glucuronide | [M-H] | 256000 | 9.47 | C21H18O12 | 461.073 | 461.0722 | -0.9 | 98.7 |
| 36 | Nodakenin | [M-H] | 696000 | 9.77 | C20H24O9 | 453.14 | 453.1396 | -1.4 | 96.5 |
| 37 | Aempferol-3-O-rutinoside | [M-H] | 775400 | 9.81 | C27H30O15 | 593.151 | 593.1504 | -1.3 | 96.2 |
| 38 | Pinoresinol-glucoside | [M-H] | 110900 | 10.18 | C26H32O11 | 519.187 | 519.1867 | -1 | 95.8 |
| 39 | Astragalin | [M-H] | 3774000 | 10.19 | C21H20O11 | 447.093 | 447.0928 | -1 | 100 |
| 40 | Naringin | [M-H] | 2630000 | 10.47 | C27H32O14 | 579.172 | 579.1718 | -0.2 | 98.3 |
| 41 | Apigenin-7-glucoside | [M-H] | 8386000 | 10.57 | C21H20O10 | 431.098 | 431.0983 | -0.2 | 97.1 |
| 42 | Hesperidin | [M-H] | 122200 | 10.8 | C28H34O15 | 609.182 | 609.182 | -0.8 | 100 |
| 43 | Dicaffeoylquinic Acid (Cynarin) | [M-H] | 11110000 | 10.81 | C25H24O12 | 515.119 | 515.1192 | -0.6 | 86 |
| 44 | Rosmarinic acid | [M-H] | 188700 | 11.03 | C18H16O8 | 359.077 | 359.0769 | -1.1 | 98.6 |
| 45 | Pratensein-7-O-glucoside | [M-H] | 456800 | 11.09 | C22H22O11 | 461.109 | 461.1084 | -1.2 | 99.3 |
| 46 | Diosmetin | [M-H] | 222200 | 11.1 | C16H12O6 | 299.056 | 299.0557 | -1.3 | 78.5 |
| 47 | Isoferulic acid | [M-H] | 46460 | 11.34 | C10H10O4 | 193.051 | 193.0505 | -0.9 | 74.1 |
| 48 | Salvianolic acid B | [M-H] | 868400 | 11.81 | C36H30O16 | 717.146 | 717.1455 | -0.8 | 98.4 |
| 49 | 7-O-beta-D-glucuronide | [M-H] | 593300 | 12.09 | C21H18O11 | 445.078 | 445.0774 | -0.6 | 99.4 |
| 50 | Ononin | [M-H] | 67880 | 12.13 | C22H22O9 | 475.125 | 475.1243 | -0.6 | 99.6 |
| 51 | Daidzein | [M-H] | 58770 | 12.33 | C15H10O4 | 253.051 | 253.0503 | -1.1 | 94.6 |
| 52 | Isoliquiritigenin | [M-H] | 85720 | 12.61 | C15H12O4 | 255.066 | 255.0661 | -0.7 | 92.3 |
| 53 | Eriodictyol | [M-H] | 865600 | 12.83 | C15H12O6 | 287.056 | 287.0558 | -1 | 96.8 |
| 54 | Luteolin | [M-H] | 2963000 | 13.02 | C15H10O6 | 285.04 | 285.0402 | -0.8 | 94.2 |
| 55 | Quercetin | [M-H] | 21610 | 13.08 | C15H10O7 | 301.035 | 301.0353 | -0.4 | 92 |
| 56 | Calycosin-7-o-glucoside | [M-H] | 6378000 | 13.73 | C22H22O10 | 491.119 | 491.1184 | -2.2 | 99.1 |
| 57 | Wogonin 7-O-glucuronide | [M-H] | 666900 | 13.83 | C22H20O11 | 459.093 | 459.0927 | -1.3 | 98.4 |
| 58 | Asperosaponin Ⅵ | [M-H] | 43930 | 14.19 | C47H76O18 | 973.501 | 973.4993 | -2.2 | 92.4 |
| 59 | Apigenin | [M-H] | 4778000 | 14.25 | C15H10O5 | 269.046 | 269.0448 | -2.8 | 93 |
| 60 | Glycyrrhizic acid | [M-H] | 1585000 | 15.11 | C42H62O16 | 821.397 | 821.3953 | -1.5 | 91.2 |
| 61 | Aurantio-Obtusin | [M-H] | 1473000 | 15.26 | C17H14O7 | 329.067 | 329.0663 | -1.3 | 96.2 |
| 62 | Astragaloside Ⅱ | [M-H] | 25790 | 15.41 | C43H70O15 | 871.47 | 871.4671 | -3 | 95.7 |
| 63 | Saikosaponin A/D | [M-H] | 224000 | 15.56 | C42H68O13 | 825.464 | 825.4631 | -1.4 | 99.4 |
| 64 | Chrysosplenetin B | [M-H] | 195700 | 15.78 | C19H18O8 | 373.093 | 373.0925 | -1 | 94.5 |
| 65 | Acacetin | [M-H] | 1626000 | 15.85 | C16H12O5 | 283.061 | 283.0608 | -1.4 | 98.4 |
| 66 | Eupatilin | [M-H] | 147400 | 16.28 | C18H16O7 | 343.082 | 343.0818 | -1.5 | 97.3 |
| 67 | Astragaloside I | [M-H] | 48500 | 16.35 | C45H72O16 | 913.48 | 913.4789 | -1.5 | 85.7 |
| 68 | Gingerglycolipid B | [M-H] | 14090 | 17.33 | C33H58O14 | 723.381 | 723.3802 | -1 | 96.3 |
